# A Prospective Study of Mortality from Cryptococcal Meningitis following Treatment Induction with 1200mg Oral Fluconazole in Blantyre, Malawi

**DOI:** 10.1371/journal.pone.0110285

**Published:** 2014-11-06

**Authors:** Katherine M. Gaskell, Camilla Rothe, Roshina Gnanadurai, Patrick Goodson, Chikondi Jassi, Robert S. Heyderman, Theresa J. Allain, Thomas S. Harrison, David G. Lalloo, Derek J. Sloan, Nicholas A. Feasey

**Affiliations:** 1 Malawi Liverpool Wellcome Trust Clinical Research Programme, University of Malawi College of Medicine, Blantyre, Malawi; 2 Liverpool School of Tropical Medicine, Liverpool, United Kingdom; 3 Department of Medicine, University of Malawi, College of Medicine, Blantyre, Malawi; 4 Queen Elizabeth Central Hospital, Ministry of Health, Blantyre, Malawi; 5 Infection and Immunity Research Centre, St. George's Medical School, University of London, London, United Kingdom; 6 Liverpool Heart and Chest Hospital, Liverpool, United Kingdom; David Geffen School of Medicine at University of California Los Angeles, United States of America

## Abstract

**Objective:**

We have previously reported high ten-week mortality from cryptococcal meningitis in Malawian adults following treatment-induction with 800mg oral fluconazole (57% [33/58]). National guidelines in Malawi and other African countries now advocate an increased induction dose of 1200mg. We assessed whether this has improved outcomes.

**Design:**

This was a prospective observational study of HIV-infected adults with cryptococcal meningitis confirmed by diagnostic lumbar puncture. Treatment was with fluconazole 1200mg/day for two weeks then 400mg/day for 8 weeks. Mortality within the first 10 weeks was the study end-point, and current results were compared with data from our prior patient cohort who started on fluconazole 800mg/day.

**Results:**

47 participants received fluconazole monotherapy. Despite a treatment-induction dose of 1200mg, ten-week mortality remained 55% (26/47). This was no better than our previous study (Hazard Ratio [HR] of death on 1200mg vs. 800mg fluconazole: 1.29 (95% CI: 0.77–2.16, p = 0.332)). There was some evidence for improved survival in patients who had repeat lumbar punctures during early therapy to lower intracranial pressure (HR: 0.27 [95% CI: 0.07–1.03, p = 0.055]).

**Conclusion:**

There remains an urgent need to identify more effective, affordable and deliverable regimens for cryptococcal meningitis.

## Introduction

Cryptococcal meningitis is the commonest cause of meningitis in adults in sub Saharan African (SSA) countries with high HIV seroprevalence [1]. The global incidence of cryptococcal meningitis was estimated at 957,900 cases/year in 2009 and 75% of cases occur in SSA [2]. In Blantyre, Malawi, *C. neoformans* was responsible for 70% of adult CSF-culture positive meningitis presenting to a tertiary referral hospital from 2000 until 2012 [3]. This burden of cryptococcal meningitis has not changed despite a highly successful national programme of antiretroviral therapy (ART) roll-out since 2004 [3].

Current gold standard induction therapy is two weeks of amphotericin B and flucytosine [4], [5]; however these drugs remain largely unavailable in SSA including Malawi [6]. Amphotericin B is not only expensive, but difficult to administer and associated with toxicities which are challenging to monitor in resource-poor settings. Consequently, high-dose oral fluconazole is widely used in SSA, but has significantly weaker early fungicidal activity than the gold standard regimen [4], [7].

We have previously reported extremely poor outcomes from cryptococcal meningitis in Blantyre, when treated with 800mg daily oral fluconazole as induction therapy [8]. In 2011 the Malawian national treatment guidelines regarding the management of cryptococcal meningitis changed, increasing the initial dose of fluconazole at treatment induction from 800mg to 1200mg daily [9]. Many African health services elected to make this change following a study that demonstrated better early fungicidal activity using 1200mg fluconazole than with 800mg [10]. We present a pragmatic, prospective observational study of clinical outcomes from cryptococcal meningitis using this dose, which is the current standard of care for many African countries.

## Methods

Queen Elizabeth Central Hospital (QECH) Blantyre is the largest government hospital in Malawi and admits approximately 10,000 adult patients per year. All patients with clinical features of meningitis undergo diagnostic lumbar puncture (LP). Inclusion criteria were unchanged from the previous study [8].

Consecutive adult patients (age ≥16) with a first presentation of cryptococcal meningitis were recruited between September 2012 and May 2013. The diagnosis was confirmed by positive India-Ink microscopy of CSF or culture-confirmed *C. neoformans* from CSF. Cryptococcal antigen testing (CrAg), quantitative cryptococcal cultures and fluconazole resistance testing were unavailable. Subjects' clinical history, including HIV diagnosis and ART history were recorded. Patients without a recent HIV test were confidentially counselled and tested. Presence of focal neurological deficit, Glasgow Coma Score (GCS) and modified Rankin score (mRS) were recorded. mRS is a 6 point disability scale (0 =  No symptoms, 1 =  No significant disability, 2 =  Minor disability, 3 =  Moderate disability, 4 =  Moderate-severe disability, 5 =  Severe disability/bed-ridden). Patients were reviewed on admission to the study, on discharge home, at four weeks and ten weeks from diagnosis.

Patients were treated according to national guidelines with 1200mg fluconazole per day for two weeks at induction followed by 400mg/day for a further 8 weeks, then lifelong secondary prophylaxis at 200mg/day [9]. A small donated supply of Amphotericin B was sporadically available for readmitted patients with evidence of fluconazole failure and patients swapped to this agent were withdrawn from the study. Patients not already receiving ART were initiated 4 weeks after diagnosis. Although national guidelines recommend daily LPs to serially reduce intracranial pressure (ICP) during early therapy, this was impossible due to staffing limitations and a lack of CSF manometry equipment. Routine practice at QECH was to undertake therapeutic LP in the event of symptoms suggesting raised ICP (e.g. severe headache).

The study endpoint was mortality at 10 weeks from diagnosis.

Statistical analysis was undertaken using “R” (version 2.15.2). Clinical parameters of study participants were compared with those from our previous study [6] by a two sample Wilcoxon test for continuous variables or a χ^2^-test for categorical variables. The study endpoint, and the relationships between prior ART or repeat LPs and mortality were assessed by survival analysis using Hazard Ratios (HRs) and Kaplan-Meier plots. The binomial exact test was used to calculate confidence intervals (CI) around death rates.

The study was prospectively approved by the University of Malawi College of Medicine Research Ethics Committee (COMREC no: P04/10/926). Informed written consent was obtained from patients to enrol in the study. Informed written consent was obtained from guardians if patients lacked mental capacity, due to advanced cryptococcal disease, to provide valid consent. The consent procedure and forms were reviewed and approved by COMREC, including the guardian consenting procedure. Two copies of the consent forms were signed per patient; one was retained by the patient and another by the study team.

## Results

58 patients were screened for enrolment, 3 patients were excluded because *C. neoformans* was isolated from blood only. Five patients were lost to follow up and therefore not included in the analysis. 3 patients were withdrawn because they were switched to Amphotericin B therapy by their physician; 2 of whom survived. Data from the remaining 47 patients is presented here; 46 were CSF culture-positive for *C. neoformans* and one patient was culture negative but India-Ink microscopy positive.

The median age was 35 years (Inter-quartile range [IQR]: 32–40 years) and 51% (24/47) were male. All patients presented with headache, the median duration at presentation was 7 days (IQR: 7–17 days). 24% (11/46) had a GCS <14/15. mRS scores showed 24/50 (51%) subjects had moderate to severe disability (grade 3–5).

All patients were HIV infected with a median CD4 count of 36 cells/µl (IQR: 17–62 cells/µl). At baseline, 45% (21/47) were taking ART for a median duration of 63 days (IQR: 21–551 days). A further 17% (8/47) commenced ART during the course of the study and 6% (3/47) had previously commenced ART but defaulted treatment.

**Figure 1:**
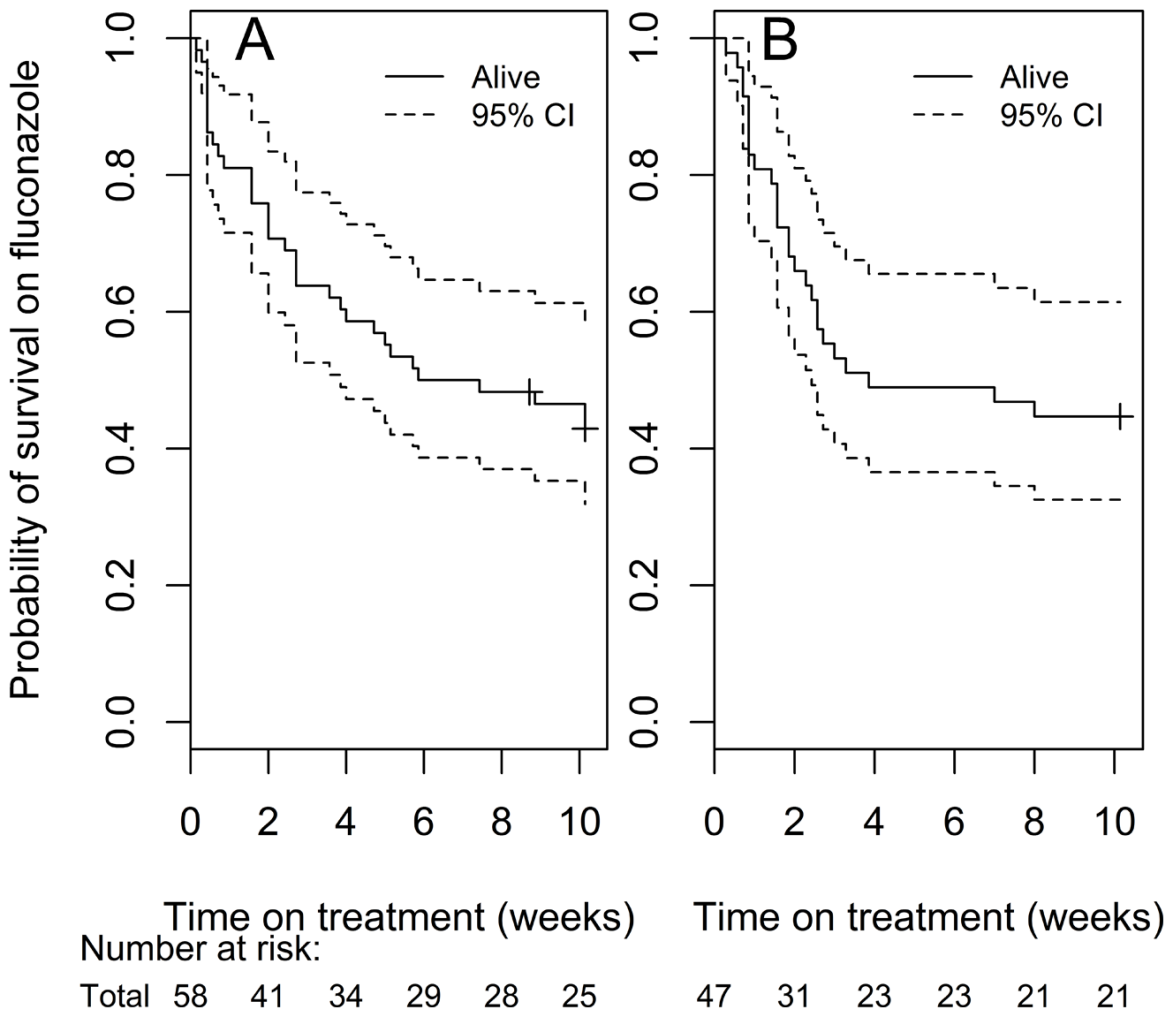
Kaplan-Meier Survival plot of patients on fluconazole. A: 800mg induction dose. B: Kaplan-Meier Survival plot of patients on fluconazole 1200mg induction dose.

Mortality at 10 weeks was 55% (26/47). The median time to death was 16 days (IQR: 7–49 days). In the previous cohort, mortality at 10 weeks amongst patients who received fluconazole 800mg/day was 57% (33/58) and median time to death was 19 days (IQR: 6–61 days). Figure 1 shows Kaplan-Meier survival plots for patients initiated on fluconazole 800mg (1A) and fluconazole 1200mg (1B); there is no difference in survival between the two induction doses. The HR for death on 1200mg vs. 800mg was 1.29 (95% CI: 0.77–2.16, p = 0.332).

The only significant clinical differences between patients in this study and the previous cohort pertained to ART; a higher proportion of patients in the current study were taking ART at enrolment and the duration of prior ART was longer (see Table 1). In the current study, there was some evidence of improved survival in patients on ART prior to enrolment (HR for death in those who presented on ART vs. those not on ART: 0.48 [95% CI: 0.21–1.07, p = 0.071]). Combining all 105 patients across both studies, mortality was not significantly affected by prior ART (HR for death: 0.84 [95% CI: 0.49–1.46, p = 0.550]). No other evidence of difference in clinical severity at presentation between the two cohorts was observed.

**Table 1:** Comparison of baseline variables between patients initiated on fluconazole 800mg and those initiated on fluconazole 1200mg.

Baseline variable	Patients initiated on *Fluconazole 800mg OD* N = 58 [8]	Patients initiated on *Fluconazole 1200mg OD* N = 47	p-value^a^
Age in years, median (IQR)	32 (29–39)	35 (32–40)	0.130
Male sex, n (%)	33 (55)	24 (51)	0.824
Headache duration in days, median (IQR)	14 (5–30)	7 (7–17)	0.508
Cranial nerve palsy/localising signs, n (%)^b^	5 (10)	12 (21)	0.225
GCS <14/15, n (%)	14 (25)	11 (24)	1
Modified Rankin Score>3/5, n (%)	24 (41)	23 (49)	0.564
HIV status known at recruitment, n (%)	35 (60)	35 (75)	0.187
CD4 count in cells/µl, median (IQR)	37 (11–58)	36 (17–62)	0.721
On ART at baseline, n (%)	13 (22)	21 (45)	0.027
Duration of prior ART, median (IQR)^c^	20 (5–67)	63 (21–511)	0.048

a Continuous variables analysed by Wilcoxon test, categorical variables analysed by χ^2^-test test.

b Includes blindness, cranial nerve palsies or focal weakness.

Repeat CSF drainage was performed on 11/23 (23%) patients in the current study and 16/58 (28%) in the previous cohort with symptoms suggestive of raised ICP. In the current study, there was weak evidence towards survival in patients who had repeat LPs (HR for death if LP repeated: 0.27 [95% CI: 0.07–1.03, p = 0.055]). When data from both studies were combined, this weak survival evidence persisted (HR for death: 0.52 [95% CI: 0.25–1.07, p = 0.077]).

This study was not designed as a prospective randomised controlled comparison two doses of fluconazole. Instead, we present prospective observational data revealing death from fluconazole in 55% (95% CI: 40–70%) patients treated with 1,200mg fluconazole and 57% (95% CI: 43–70%) patients treated with 800mg fluconazole. Both studies indicate that cryptococcal meningitis therapy based on induction with oral fluconazole, whether at 800 or 1200mg/day achieves unacceptably poor outcomes.

## Discussion

A Ugandan study of fluconazole demonstrated higher EFA in CSF at a daily dose of 1200mg than 800mg without increased toxicity [10], and it was hoped that this would translate into improved clinical outcomes. However, a more recent pharmacokinetic-pharmacodynamic (PK-PD) model of treatment with 1200mg fluconazole predicted that only 67% of patients on this dose will obtain adequate CSF drug concentrations to achieve fungal stasis [11]. In our study, 10-week mortality from cryptococcal meningitis in Malawi has not improved following an increase in the induction dose of fluconazole from 800mg to 1200mg. The highest mortality occurred in the initial two weeks, suggesting that rapid fungal clearance is essential for a good clinical outcome.

Expanded access to ART in Malawi [12] meant that more patients had received ART before recruitment to the current cohort than in our previous study of fluconazole 800mg/day [8]. This raises the possibility that Immune Reconstitution Inflammatory Syndrome (IRIS) masked a benefit from the higher dose of fluconazole, however in the current study, there was slightly higher survival in patients on ART and in a combined analysis of both studies the effect of prior ART was non-significant. Secondly IRIS is unlikely as these patients were culture positive with CD4 counts <50 cells/uL at presentation. Overall, these data do not support a negative confounding effect from IRIS.

Despite improved ART provision, there remains a large population with advanced HIV in Blantyre, and there is an urgent need to protect them from cryptococcal meningitis. General strategies include earlier HIV diagnosis and treatment. A specific approach with increasing evidence of cost-effectiveness is CrAg screening and treatment of asymptomatic antigenaemia in the ART clinic [13,14,15].

Several studies have reported that serial LPs to lower ICP during treatment of cryptococcal meningitis may improve outcomes [16], [17]. Although Malawian national guidelines suggest performing daily LPs during early therapy [9], there is neither adequate staffing nor the equipment for this to be possible. Without CSF manometers we could only repeat LPs on patients with symptoms of raised ICP. There was weak evidence of improved survival in this group. Importantly the individuals selected for additional procedures had the worst initial symptoms; these data support the importance of therapeutic CSF drainage. The benefit of serial LPs may be even greater if they are routinely guided by CSF pressure measurement.

The current and previous study recruited patients with similar characteristics in the same setting, however they have limitations; the studies were undertaken sequentially, not as part of a combined randomised controlled trial. Furthermore, there was some loss to follow up and it was different to monitor adherence to therapy. Serial CD4-counts and HIV-viral loads were unavailable preventing a formal diagnosis of IRIS. Nevertheless, the studies provide important information on the treatment of cryptococcal meningitis from an authentic high-burden setting.

Oral fluconazole is currently the only routinely available therapy in much of SSA, but mortality rates are unacceptably high. An increase in the recommended induction dose from 800mg to 1200mg daily has had no impact on clinical outcomes in Malawi. There are on-going studies with even higher doses of fluconazole, (ACTG study: http://clinicaltrials.gov/show/NCT00885703) and of novel therapeutic strategies (“Advancing cryptococcal meningitis treatment in Africa” [ACTA] ISRCTN: 45035509). Whilst the outcome of these studies is awaited, there remains an urgent need for expanded access to amphotericin B and flucytosine across SSA.
